# Barbamide Displays Affinity for Membrane-Bound Receptors and Impacts Store-Operated Calcium Entry in Mouse Sensory Neurons

**DOI:** 10.3390/md21020110

**Published:** 2023-02-02

**Authors:** Andrea Hough, Connor Criswell, Asef Faruk, Jane E. Cavanaugh, Benedict J. Kolber, Kevin J. Tidgewell

**Affiliations:** 1 Graduate School of Pharmaceutical Sciences, Duquesne University, Pittsburgh, PA 15282, USA; 2 Center for Advanced Pain Studies, Department of Neuroscience, University of Texas at Dallas, Richardson, TX 75080, USA

**Keywords:** cyanobacteria, kappa opioid receptor, sigma-2 receptor, TMEM97, cytotoxicity, barbamide, neuronal activation, SOCE

## Abstract

Marine cyanobacteria are a rich source of bio-active metabolites that have been utilized as leads for drug discovery and pharmacological tools for basic science research. Here, we describe the re-isolation of a well-known metabolite, barbamide, from Curaçao on three different occasions and the characterization of barbamide’s biological interactions with targets of the mammalian nervous system. Barbamide was originally discovered as a molluscicidal agent from a filamentous marine cyanobacterium. In our hands, we found little evidence of toxicity against mammalian cell cultures. However, barbamide showed several affinities when screened for binding affinity for a panel of 45 receptors and transporters known to be involved in nociception and sensory neuron activity. We found high levels of binding affinity for the dopamine transporter, the kappa opioid receptor, and the sigma receptors (sigma-1 and sigma-2 also known as transmembrane protein 97; TMEM97). We tested barbamide in vitro in isolated sensory neurons from female mice to explore its functional impact on calcium flux in these cells. Barbamide by itself had no observable impact on calcium flux. However, barbamide enhanced the effect of the TRPV1 agonist capsaicin and enhanced store-operated calcium entry (SOCE) responses after depletion of intracellular calcium. Overall, these results demonstrate the biological potential of barbamide at sensory neurons with implications for future drug development projects surrounding this molecule.

## 1. Introduction

**Figure 1:**
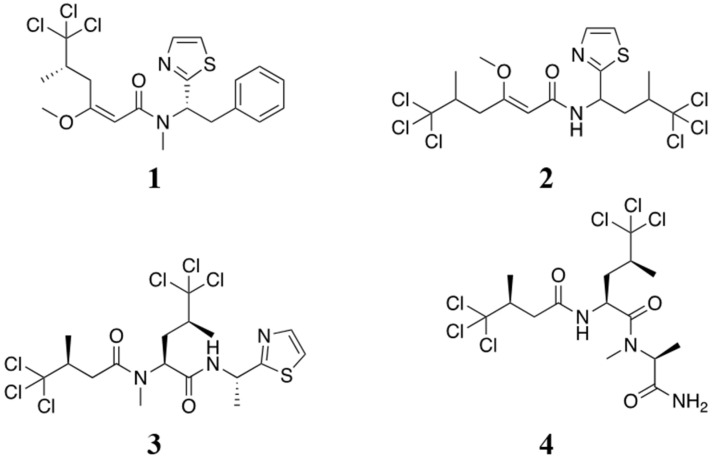
Structure of barbamide (**1**) and analogs: barbaleucamide A (**2**), dysidenin (**3**) and dysidenamide (**4**).

Originally isolated in 1996 by the Gerwick lab, barbamide (**1**; Figure 1) was reported to be a molluscicidal agent [1]. Since that original report, barbamide has been re-isolated from other cyanobacterial collections suggesting broad utility as a signaling molecule for filamentous marine cyanobacteria [2]. There have been a number of studies investigating the biosynthesis of barbamide (**1**) [3] and its analogs, the barbaleucamides A (**2**) and B [4], dysidenin (**3**) [5], and dysidenamide (**4**) [6]. While its biosynthesis has been well studied, the activities of barbamide and its analogs have not been extensively described. Aside from mild toxicity, no strong cytotoxic or other potent pharmacological or ecological activities have been reported for this compound. In this manuscript, we explore the activities of barbamide against targets not previously explored. We have focused our efforts on understanding the bioactivity of barbamide by investigating the interactions of this molecule with membrane receptors and its effects on neuronal activity and function. 

## 2. Results

**Figure 2:**
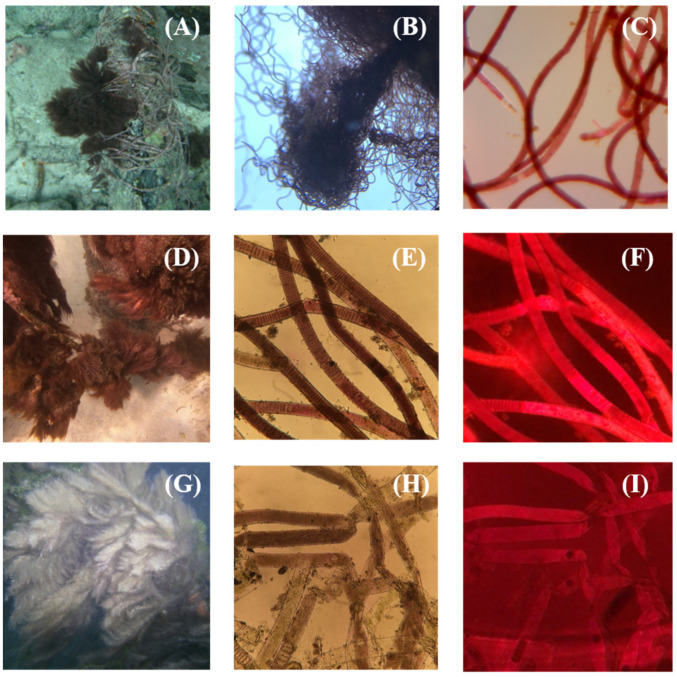
Photos of barbamide-containing marine samples collected in Curaçao. (**A**–**C**) Images of DUQ0029 collected at CARMABI Station in 2015. (**A**) Live sea-life image. (**B**) 7.5× light microscopy image. (**C**) 10× light microscopy image. (**D**–**F**) Images of DUQ0032 collected at CARMABI Station in 2018. (**D**) Live sea-life image. (**E**) 10× light microscopy image. (**F**) 10× epi-fluorescent microscopy image of specimen. (**G**–**I**) Images of DUQ0037 collected at Spanish Waters in 2018. (**G**) Live sea-life image. (**H**) 10× light microscopy image. (**I**) 10× epi-fluorescent microscopy image of specimen.

A red-purple *Moorea* was collected under a dock at the CARMABI Research Station in Curaçao in 2015 (Figure 2 A–C). The sample (DUQ0029) was extracted exhaustively in 2:1 DCM:MeOH and the extract was then subjected to silica gel chromatography using a stepwise gradient of hexane/EtOAc and EtOAc/MeOH to produce nine fractions. All fractions were then screened for binding affinity against a panel of central nervous system receptors (Table 1).

Fraction DUQ0029D (110 mg), which eluted in 2:3 Hexanes:EtOAc, showed binding affinity for multiple serotonin (5-HT) receptors, the kappa opioid receptor (KOR), and both sigma receptors (Table 1). The fraction was then subjected to reversed-phase flash chromatography to yield a pure compound which was determined to be barbamide (43.3 mg). The pure compound was re-screened against the same panel of receptors as the original fractions (Table 2). Secondary screening with targets where the percent inhibition in primary screening was greater than 50% was conducted. Secondary screening showed that barbamide displayed affinity for a number of targets: KOR (K_i_ = 79.14 nM), sigma-1 (K_i_ = 2256 nM) and sigma-2 (*TMEM97*) (K_i_ = 2640 nM). Additionally, barbamide showed affinity for the dopamine D3 receptor (D_3_R) (K_i_ = 446 nM) and the dopamine transporter (DAT) (K_i_ = 3100 nM). 

The halogenation of barbamide results in a unique isotope pattern in the mass spectral chromatogram, making targeted re-isolation facile. Since isolating barbamide from DUQ0029, a targeted screen of our fraction library was conducted and we were able to re-isolate barbamide from two additional samples. The first sample, DUQ0032, was a red-purple *Moorea* collected from the same location at CARMABI Station in Curaçao in 2018, 3 years after our original collection (Figure 2D–F). The second sample, DUQ0037, was a red-brown *Moorea* growing in the mangroves of “Spanish Waters” in Curaçao (Figure 2G–I). Interestingly, we collected this near (less than 1 km from) Barbara Beach, Curaçao, which was the collection site of the sample from which barbamide was originally isolated by the Gerwick lab [1]. A broad receptor screen was conducted on the fractions of these extracts, and pure barbamide was isolated from DUQ0029D. Each of the barbamide-containing fractions had similar binding profiles against the sigma-2 receptor and KOR (Table 2), showing the reproducibility of the results and findings. With an understanding of the receptors that barbamide shows affinity for and could modulate the activity of, barbamide was then submitted to in vitro biological assays to evaluate its cytotoxicity and cellular function.

We initially investigated whether barbamide demonstrated any cytotoxicity against human-derived cells in order to determine if it could be useful as a membrane receptor modulator without causing off-target cell death. Because barbamide and its parent fraction showed affinity for the sigma-1 receptor and the sigma-2 receptor (aka *TMEM97* [7]) both known to be up-regulated in some TNBC and other cancer cell lines [8,9], we wanted to investigate its cytotoxic effects on these cells. Barbamide was screened against MDA-MB-231 and BT-549 TNBC cells, along with MCF-7 breast cancer cells and HEK-293 cells using an MTT assay but did not significantly decrease cell viability at concentrations required for receptor activation (Table 3). Briefly, 3-(4,5-dimethylthiazol-2-yl)-2,5-diphenyltetrazolium bromide (MTT) is converted from a yellow tetrazole containing compound to a purple formazan containing compound in living cells, so the assay uses MTT as a colorimetric marker of cell viability. 

**Figure 3:**
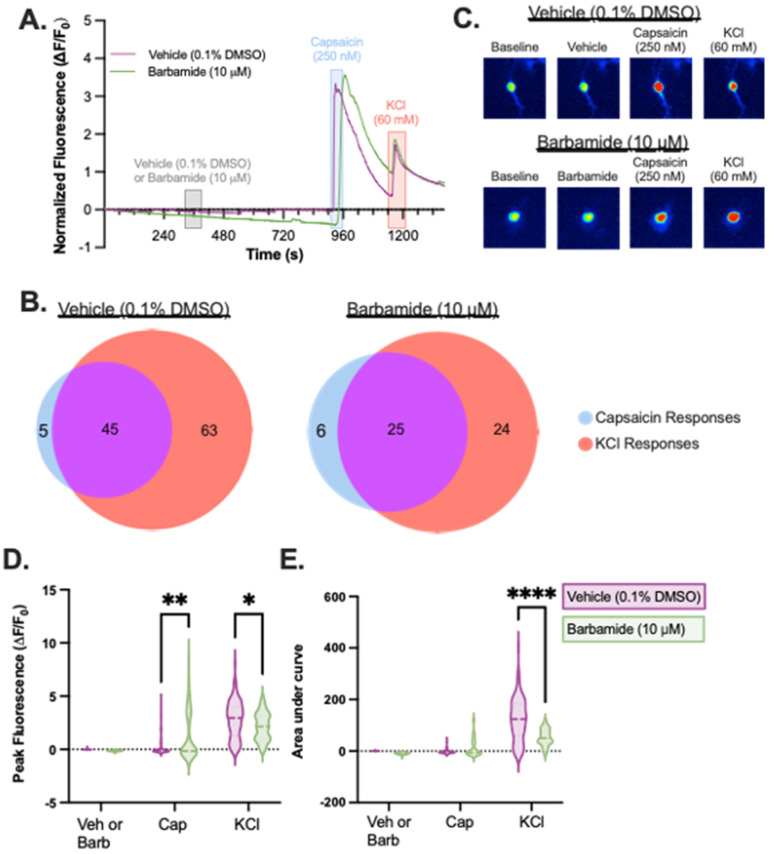
Effect of barbamide on capsaicin response in dorsal root ganglion sensory neurons. Primary mouse DRG expressing the Ca^2+^ sensitive GcaMP6f were subjected to barbamide or vehicle to test its effect on the capsaicin excitatory response. (**A**) Representative calcium imaging fluorescent traces of two neurons, one treated with vehicle and one treated with barbamide (10 mM) (**B**) The distribution of recorded cells that exhibited a change in calcium activity in response to Ca^2+^ and/or KCl with prior treatment with either vehicle or barbamide. (**C**) Representative images showing calcium response to treatment. (**D**) Barbamide increased the effect of capsaicin on calcium transients as measured by peak fluorescence responses. Peak responses to KCl were dampened by prior Barbamide treatment. (**E**) AUC for the KCl response. 2-Way ANOVA followed by Sidak’s multiple comparison tests. * *p* < 0.05; ** *p* < 0.01; **** *p* < 0.0001; Barbamide = 55 cells; Vehicle = 113 cells.

After confirming that barbamide showed minimal cytotoxic effects, we shifted our focus to look at its ability to alter neuronal function. Because sigma-2/TMEM97 receptors have recently been shown to modulate pain-like activity in rodents [10,11,12,13], we measured evoked calcium signals in peripheral sensory neurons with and without drug application in vitro (Figure 3A–E) to determine possible pain-modulating effects of barbamide. We evoked calcium transients in mouse dorsal root ganglion (DRG) neurons expressing a genetically encoded calcium indicator (GCaMP6f). Barbamide alone had little observed impact on DRG calcium responses. However, compared to vehicle, barbamide significantly increased the maximum fluorescence intensity of the cells during their responses to capsaicin (*p* < 0.0001, two-way ANOVA followed by Sidak’s multiple comparison tests; Figure 3D). While responses to capsaicin were sensitized, the subsequent response to depolarization by KCl was diminished in barbamide-treated cells both in the maximum fluorescence intensity and in the area under the curve of the fluorescent traces (Figure 3E). 

**Figure 4:**
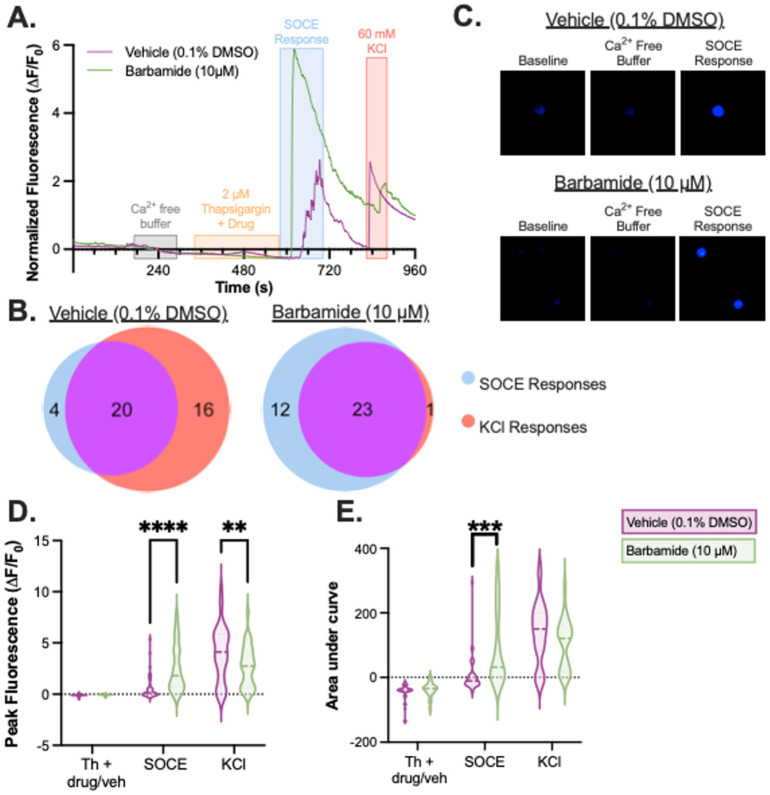
Effect of barbamide on SOCE in dorsal root ganglion sensory neurons. Primary DRG expressing the Ca^2+^ sensitive GCaMP6f were subjected to barbamide or vehicle to test its effect on the SOCE response in the context of the SERCA inhibitor thapsigargin. (**A**) Representative calcium imaging fluorescent traces of two neurons, one treated with vehicle + thapsigargin and one treated with barbamide (10 µM) + thapsigargin. (**B**) The distribution of recorded cells that exhibited a change in calcium activity in response to Ca^2+^ or KCl. (**C**) Barbamide + thapsigargin enhanced the SOCE effect (bottom images) compared to vehicle + thapsigargin (upper images) in both the (**D**) max signal during the SOCE response and the (**E**) AUC for the SOCE response. 2-Way ANOVA followed by Sidak’s multiple comparisons test. ** *p* < 0.01; *** *p* < 0.001; **** *p* < 0.0001; Barbamide = 36 cells; Vehicle = 40 cells.

We also investigated barbamide’s effect on the DRG neurons’ store-operated calcium entry (SOCE) response. The SOCE response was induced in the DRG neurons by submersing the cells in a Ca^2+^-free buffer and using the sarco/endoplasmic reticulum Ca^2+^ ATPase (SERCA) inhibitor thapsigargin to deplete ER Ca^2+^ stores. Thapsigargin treatment was combined with either vehicle or barbamide to evaluate the impact of treatment on the mechanisms of SOCE development (Figure 4). Upon the reintroduction of Ca^2+^-containing buffer (no vehicle or barbamide) to the recording chamber, an influx of calcium across the plasma membrane was observed. Compared to vehicle, barbamide significantly increased both the maximum fluorescence intensity and overall calcium flux, suggesting an enhancement of SOCE homeostatic responses in response to barbamide (*p* < 0.0001, two-way ANOVA followed by Sidak’s multiple comparison tests; Figure 4D–E).

## 3. Discussion

Barbamide is a known cyanobacterial metabolite that has been isolated from a variety of cyanobacterial species and collection sites [2]. Although barbamide and its analogs have been repeatedly isolated, the activity of these compounds is not well explored. Our initial interest in this compound arose when it was isolated from a cyanobacterial fraction that demonstrated sigma-1 receptor, sigma-2/TMEM97 receptor, DAT, and KOR affinity. Our research has focused on discovering cyanobacterial secondary metabolites with CNS modulatory activity [14,15,16]. We used barbamide as a tool to demonstrate the in vitro activity of a cyanobacterial compound on neuronal cells. Barbamide represents a useful tool because it can be easily re-isolated to obtain more material, it shows limited cytotoxicity, and previously had no reported CNS activities. We have been able to show that barbamide has affinity for CNS receptors and modulates of Ca signaling in in vitro systems. While the observed cellular actions of barbamide are not ideal for use as a drug, its structure and small molecular size make it an interesting starting point for future hit-to-lead optimization. 

We started by testing barbamide for cytotoxicity because of previous reports of molluscicidal activity and its affinity for sigma receptors which have been shown to be upregulated in rapidly proliferating cancer cell lines [8,9]. Barbamide did not demonstrate significant cytotoxicity in the cell lines tested, including three breast cancer cell lines and a non-cancerous cell line. This meant that as we explored the activity of barbamide within the CNS, it is less likely that any CNS activity would be accompanied by off-target cytotoxic effects to non-cancerous cells. To look for CNS activity, we utilized the Psychoactive Drug Screening program to evaluate our cyanobacterial fractions for affinity for a panel of CNS receptors, transporters, and ion channels involved in CNS dysfunction and disease. The panel screened includes major GPCR targets (dopaminergic, serotonergic, opioid, cannabinoid, etc.), monoamine transporters (DAT, NET, SERT), and other well-known targets involved in pain and other CNS disorders. This screen starts with testing a single concentration in radioligand binding assays as a primary screen, and then fractions and compounds which show greater than 50% inhibition of binding are subjected to secondary screening to determine an IC_50_ (in the case of fractions where we do not know the molecular composition) or a K_i_ (for pure compounds where we know the molecular mass). Fraction-level affinity can be misleading due to the potential presence of multiple compounds with different activity, which is where the affinity and activity of the pure compound become more important. 

Using mouse dorsal root ganglion neurons, we were able to show for the first time biological effects of barbamide outside of a generic cellular toxicity phenotype. First, we found that there was no effect of barbamide on Ca^2+^ flux in mouse DRG when directly applied in vitro. This supports the notion that barbamide may be working through a modulatory role rather than as a direct activator of Ca^2+^ channels or other cation channels. However, despite no observed direct effects of barbamide on Ca^2+^ flux, we did see an enhancement of Ca^2+^ flux upon subsequent application of the TRPV1 agonist capsaicin. This effect points to a sensitization of the DRG by barbamide. Second, we found a similar enhancing effect of barbamide on the SOCE response after intracellular Ca^2+^ depletion with the SERCA inhibitor thapsigargin. 

Based on hits from the receptor affinity screening of barbamide, we found that the three most interesting targets for further exploration are DAT, KOR, and the sigma receptors. Based on previous research with DRG, the three most likely mediators of the effects seen here are either the KOR or the sigma receptors, as DAT is not expressed at meaningful levels in either mouse or human DRG [17,18], while both KOR and sigma receptors are expressed. 

KOR activation in mouse DRG tends to inhibit excitability of both peptidergic nociceptors and low-threshold mechanoceptors [19]. Activation of KOR pre-synaptically in nociceptors hyperpolarizes neurons and reduces release of excitatory neurotransmitters into the dorsal horn of the spinal cord [19]. Peripherally restricted KOR agonists (e.g., nalfurafine) inhibit chemical pain and mechanical hypersensitivity following peripheral injury [19]. There have never been any reports of KOR agonists or antagonists on SOCE responses in DRG or central neurons although KOR agonists inhibit Ca^2+^ flux in human DRG in the presence of high extracellular KCl. It is unlikely that the effects observed for barbamide were mediated by KOR. Since we saw excitation rather than inhibition with barbamide, this cyanobacterial metabolite would have to be acting as an antagonist to KOR. However, an antagonist of KOR would not be expected to have an effect in the absence of KOR ligands and would be unlikely to influence Ca^2+^-mediated influx caused by activation of TRPV1 with capsaicin. It is possible that barbamide is acting as an inverse agonist for KOR, but we would have expected enhanced Ca^2+^ flux during KCl application; we saw a decrease in Ca^2+^ flux during depolarization. On the other hand, it is possible that the barbamide effects were mediated by one or both of the sigma receptors, sigma-1 or sigma-2/TMEM97. 

Our first fraction and pure barbamide screening suggested binding of barbamide at the sigma-1 receptor. While we did not show robust sigma-1 binding in our later re-collections of barbamide-containing fractions and barbamide itself only showed 50% primary binding in PDSP screening, it is possible that some of the effects observed from barbamide were mediated by this receptor. The sigma-1 receptor has a well-described role in pain with potential for therapeutic targeting [20]. Sigma-1 is also known to modulate sensory neurons, including through its binding to TRPV1 [21,22]. Based on our Ca^2+^ results, a sigma-1-mediated effect of barbamide would likely have occurred agonistically. Agonists of sigma-1 reduce KCl depolarization effects through plasma membrane voltage-gated Ca^2+^ channels rather than through modulation of intracellular Ca^2+^ stores. Such a reduction in KCl depolarization fits with our observations. However, the notion that sigma-1 affects sensory neurons primarily through the modulation of voltage-gated Ca^2+^ channels does not fit as well with our observed effects of barbamide on SOCE [23].

The other sigma receptor that hit across all of our fractions and with isolated barbamide was sigma-2. The molecular identification of the sigma-2 receptor as TMEM97 was not made until 2017, 20 years after sigma-1’s identification. Further, the crystal structure of TMEM97 was only published in 2021 [7,10]. TMEM97 is in the endoplasmic reticulum, lysosomes, and plasma membrane [24,25] and is thought to participate in cholesterol homeostasis [26,27,28] and Ca^2+^ signaling. Since 2017, sigma-2/TMEM97 has been investigated as a promising drug target for pain using both new and older ligands. Sigma-2/TMEM97 has been described in multiple cell types for its influence on SOCE [29,30]. Sigma-2/TMEM97 silencing or overexpression has been shown to increase or decrease, respectively, the SOCE calcium channel Orai1 [30]. One of the mechanisms of sigma-2/TMEM97 on Ca^2+^ is through cholesterol homeostasis, which has the potential to alter properties of membrane channels such as TRPV1 [31]. TRPV1 is directly inhibited by cholesterol binding [31] and TMEM97 appears to regulate SOCE through a reduction in cholesterol content, leading to enhanced function of Ora1 with subsequent increases in Ca^+2^ influx. Thus, a parsimonious explanation for both our observed effects of barbamide on capsaicin/TRPV1 Ca^2+^ movement and thapsigargin-induced SOCE is through the interactions of barbamide with sigma-2/TMEM97. In this model, which will need to be explored in the future, barbamide, through sigma-2/TMEM97, leads to a rearrangement of cholesterol, ultimately leading to an enhancement of cellular excitability. Future studies will be necessary to clearly determine the role of sigma-1 vs. sigma-2/TMEM97 in the observed effects of barbamide.

Overall, based on the cellular responses to barbamide observed in this study, we would predict that barbamide would enhance, not inhibit, the pain-producing effects of capsaicin in intact animals. Although not tested in this manuscript, such a systems-level study would promote the potential of barbamide, and cyanobacterial metabolites in general, to modify nociceptor activity. There is considerable interest in the development of novel non-opioid analgesics for both acute and chronic pain. Although unmodified barbamide would not be a good candidate as an analgesic, it may serve as an interesting scaffold for future medicinal chemistry optimization through side chain and functional group modification, depending on the target of interest. This is speculative since barbamide was only tested in vitro in the present manuscript. Furthermore, the PDSP screening captured potential targets that barbamide might act through, but other receptors or transporters may play a role in the in vitro results observed. From a drug discovery perspective, the focus here on in vitro testing could be viewed as a limitation and validation of potential in vivo effects matching the effects in DRG would be essential before moving into drug development. 

## 4. Materials and Methods

### 4.1. General Experimental Procedures 

NMR spectra were recorded with CDCl3 (δC 77.2, δH 7.26) as internal standards on a Bruker 500 MHz spectrometer equipped with a 5 mm PATXI 1HD/D-13C/15N Z-GRD Probe, operating at 499.7 MHz for 1H and 125.7 MHz for 13C (Supplementary Materials). Mass spectra were acquired with a benchtop Advion mass spectrometer. HPLC separation was carried out using a Dionex Ultimate 3000 pump system with UV detection using HPLC-grade solvent. Accelerated chromatographic isolation was carried out on a Biotage Isolera One system with UV detection using HPLC-grade solvent. Solvents were evaporated on a Heidolph rotary evaporator.

### 4.2. Collection

- DUQ0029: A red-purple *Moorea* was collected from under a dock at CARMABI Station in Curaçao (GPS Coordinates: 12°07′23.3″ N 68°58′08.9″ W) on 6 June 2015.
- DUQ0032: A red-purple *Moorea* was collected from under a dock at CARMABI Station in Curaçao (GPS Coordinates: 12°7′20.6″ N, 68°58′7.572″ W) on 4 December 2018.
- DUQ0037: A red-brown *Moorea* was collected from shallow (0–5 ft) mangroves of Spanish Waters in Curaçao (GPS Coordinates: 12°4′48.648″ N, 68°50′40.668″ W) on 7 December 2018.

All samples were stored in isopropanol/seawater at reduced temperature until extraction. In the field (at CARMABI Research Station), a small sample was removed from the sea water, lightly dissected, and imaged at 1×–10× using light and epi-fluorescent microscopy (using a TRITC filter). 

### 4.3. Extraction and Isolation

DUQ0029 was extracted with DCM-MeOH (2:1) to a constant mass of 0.86 g and given the sample ID of DUQ0029. The crude extract was fractionated by silica gel chromatography using a stepwise gradient starting from 100% hexanes to 100% ethyl acetate to 100% methanol to yield nine fractions. Fraction DUQ0029D (110 mg), which eluted in 2:3 hexanes:EtOAc, was subjected to additional fractionation using reversed-phase flash chromatography (50% ACN/H20 for 30 mL, 50–68% ACN for 54 mL, 86% for 15 mL, 68–91% for 69 mL, then 100% for 63 mL). Fraction DUQ00029Di11-14, which eluted in 77% ACN, contained pure barbamide (43.3 mg).

Samples DUQ0032 and DUQ0037 were extracted using the same method as DUQ0029. Both extracts were fractionated by silica gel chromatography using a stepwise gradient of 80% hexanes:20% ethyl acetate to 100% ethyl acetate to 100% methanol to yield nine fractions. Fractions DUQ0032F3 and DUQ0037F3 eluted in 100% ethyl acetate. These fractions were subjected to reversed-phase HPLC (Phenomenex, Synergi-fusion 4μ, 150 × 10 mm) using a linear gradient of acetonitrile–H_2_O (50% acetonitrile for 5 min, then 50–100% acetonitrile in 25 min and then 100% acetonitrile for 5 min). Barbamide eluted at 17.1 min (74.3% acetonitrile: 25.7% water)

**Barbamide (1)**: colorless oil; the 1H-NMR results were comparable to previously reported literature values. ESI-MSm/z 483.6 calcd. for C_20_H_23_Cl_3_N_2_O2S [M + Na].

### 4.4. Cytotoxicity Assays

Cell Culture: MDA-MB-231 (ATCC, Manassas, VA) were cultured in DMEM:F-12 (1:1). BT-549 and MCF-7 (ATCC) cells were cultured in RPMI media supplemented with 5% FBS (Gibco, Thermo Fisher Scientific, Waltham, MA, USA). HEK293 cells were cultured in DMEM supplemented with 5% FBS.

Cells were plated in 96-well TPP culture plates (MidSci, St. Louis, MO, USA) at a density of 5×10^3^ cells per well. Cells were allowed to attach overnight. The cells were treated with barbamide or vehicle for 72 h with 5% FBS stimulation. DMSO was used as a vehicle control. After treatment, 10 µL MTT (Sigma, St. Louis, MO, USA) was added to each well (0.5 mg/mL final concentration) and the plates were incubated for 3 h (5% CO_2_ and 37 °C). The medium was removed and the MTT-formazan crystals were dissolved with 100 µL DMSO per well. The absorbance was measured at 570 nm with a VICTOR^3^ 1420 multilabel counter (Perkin Elmer, Waltham, MA, USA). Three wells were analyzed for each condition, and wells containing medium-MTT only (no cells) and medium-MTT (DMSO + 5% FBS) were used as controls. Results were normalized to the DMSO + 5% FBS group.

### 4.5. Animals

All animal experimental procedures were conducted according to protocols approved by the Institutional Animal Care and Use Committee of the University of Texas at Dallas (Protocol 20-04 - Approval 14 May 2020). Genetically encoded Ca^2+^ indicators (GECIs) for high-fidelity Ca^2+^ imaging in excitable cells are used to study neural circuitry in the brain and peripheral nervous system. When paired with the appropriate promoter, murine models express GECIs in specific neural subtypes or in all neurons. For use in dorsal root ganglia (DRG), we crossed a C57BL/6J mouse strain with a plasma membrane-targeted GCaMP6f conditional allele (Jackson 021405) to a nestin promoter-driven Cre recombinase (nestin-Cre)-expressing C57BL/6J mouse strain (Jackson 003771). The nestin-Cre was chosen because it is a promoter for a type VI intermediate filament prominent in neurons. Mice heterozygous for both the GCaMP6f conditional allele and the nestin-Cre are used to achieve expression of GCaMP6f in DRG. A total of 2 female adult (aged more than two months) *Nes-Cre/+;GCaMP6f/+* mice were used in this study.

### 4.6. Ex vivo DRG Preparation

Animals were decapitated, and the spinal column was dissected out and submerged in a dish filled with ice-cold Ca- and Mg-free HBSS medium (Gibco 14170112) containing 10 mM HEPES (Gibco 15630080). The whole dissection procedure was performed in less than 10 min. Under a dissecting microscope, excess tissue was removed, and the spinal column was bisected lengthwise before being transferred to a dish filled with ice-cold Hibernate-A medium (Gibco A1247501). A dorsal root ganglionectomy was performed and surgical scissors were used to trim the remaining nerve roots and connective tissue from the ganglia. All cleaned ganglia were collected into a sterile 5 mL snap cap tube containing 3 mL 37 °C HBSS/HEPES with 45 added units papain (Worthington Biochemical LS003126) and 4.5 mg collagenase from *Clostridium histolyticum* (Sigma-Aldrich C6885), then incubated at 37 °C for 30 min in 5% CO_2_.

After the incubation, the DRG were rinsed four times with 37 °C HBSS/HEPES. After the rinses, the DRG were resuspended in sterile-filtered 37 °C Neurobasal-A medium (Gibco 10888022) supplemented with 5% heat-inactivated fetal bovine serum (Rockland FBS-01-0500), 1× B-27 supplement (Gibco 17504044), 50 U/mL penicillin–streptomycin (Gibco 15070063), and 2 mM GlutaMAX (Gibco 35050061). This Neurobasal-A Complete solution was supplemented with 10 μg/mL DNase I (Roche 11284932001) to assist with dissociation and the DRG were triturated with progressively smaller fire-polished borosilicate glass pipettes until they had dissociated into a single-cell suspension. The dissociated neurons were pipetted through a 40 μm nylon cell strainer (Falcon 352340) and plated in 24-well plates containing acid-washed glass coverslips (Fisher 12-545-80P) coated with sterile 0.1 mg/mL poly-D-lysine (Sigma-Aldrich P7405). The neurons were returned to the incubator for 2–4 h to allow proper adherence to the coverslips; then, 400 mL of additional Neurobasal-A Complete solution was added to each well. The experiments were conducted exclusively on cells cultured for 16–30 h.

### 4.7. Calcium Imaging Solutions

All experimental solutions were adjusted to pH 7.38. The extracellular recording solution (ERS) contained (in mM): 129 NaCl, 5 KCl, 2.6 CaCl_2_, 1 MgCl_2_, 31 glucose, and 10 HEPES. The calcium-free extracellular solution (Ca-ERS) contained no added calcium, 3 mM MgCl_2_, and 2 mM EGTA to chelate ambient calcium.

Drug solutions for all experiments were delivered to the cells using a pressurized multi-valve perfusion system (Warner Instruments VPP-6-6). The 5 mM capsaicin (Sigma-Aldrich M2028) stock was prepared in ethanol plus 0.1% ascorbic acid (Sigma-Aldrich A5960) to help maintain potency, and aliquots were stored at −20 °C. The 250 nM capsaicin and 60 mM KCl working solutions were prepared in extracellular recording solution on the day of recording. The 10 mM thapsigargin (Tocris 1138) and 10 mM barbamide (DUQ0029D) stocks were prepared in DMSO, and aliquots were stored at −20 °C. For working solutions, the thapsigargin was diluted to 2 μM and the barbamide to 10 μM in calcium-free extracellular solution on the day of recording. 

### 4.8. Intracellular Calcium Imaging

For imaging, the slips were placed in a glass-bottom recording chamber on the stage of an upright microscope (Olympus BX51WI) and superfused with extracellular recording solution (∼2 mL/min). The excitation light was generated by an LED light source (X-Cite Xylis XT720S) and passed through an FITC filter. For GFP, the excitation wavelength was at 480 nm, and the fluorescence emission was at 510 nm. A 10×/0.30 W water-immersion lens (Olympus UMPlanFl) was used for neuronal imaging. Images were acquired by an Orca-Fusion Digital CMOS Camera (Hamamatsu C14440-20UP) at a rate of 1 frame/s with a resolution of 2304 × 2304 pixels, which corresponded to an image plane of 1.5 × 1.5 mm. Fluorescent activity representing calcium transients for each region of interest (ROI) and background (∆F/F_0_) were recorded using the cellSens Dimension imaging software (Olympus, Shinjuku, Tokyo, Japan). 

Two different experiments were completed. **(1)** Evaluation of the effect of barbamide on native Ca^2+^ flux and the cellular response to the TRPV1 ligand capsaicin. Neuronal responses were recorded while cells were exposed to a continuous flow of the following solutions (in sequential order): 2 min ERS, 1 min barbamide (10 μM) or vehicle (ERS + 0.1% DMSO), 8 min ERS, 30 s capsaicin (250 nM), 2 min ERS, and 1 min KCl (60 mM). Ca^2+^ transients were induced through application of barbamide, the TRPV1 agonist, capsaicin, or KCl. Neurons with an increase in calcium signaling in response to either capsaicin and/or KCl were selected for further analysis (Figure 3A,B). **(2)** Evaluation of the effect of barbamide on store-operated calcium entry (SOCE) responses. The following solutions were applied in sequential order during recording: 1 min ERS, 2 min Ca-ERS, 1 min barbamide (10 μM) or vehicle (ERS + 0.1% DMSO) and thapsigargin (2 μM), 3 min incubation (flow was turned off but not drained), 2 min ERS, and 1 min KCl (60 mM). Physical testing was completed unblinded to treatment for all coverslips. Neurons that displayed the SOCE response or showed an increase in calcium signaling in response to the KCl application afterwards were selected for further analysis (Figure 4A,B).

### 4.9. Statistical Analysis

Analysis of the raw fluorescent traces from cellSens was completed agnostic to treatment using a custom script in MATLAB R2022b (Mathworks). In this script, the fluorescent signal for each cell is calculated by subtracting the background fluorescence from the raw signal at each time point. The signals are then partitioned into various timeframes based on which solution is flowing into the imaging chamber at the time. For each cell, both the maximum fluorescent signal and area under the curve (AUC) are calculated, using the “max” function and the “trapz” function respectively, for each timeframe. The ΔF/F_0_ was quantified as the difference between fluorescence intensity and the averaged baseline fluorescence intensity divided by the baseline.

Further data processing, analysis, and visualization was done using Prism 9.4.1 (GraphPad Software, San Diego, CA, USA). Repeated measures two-way ANOVAs with post-hoc Sidak’s multiple comparisons were used to compare the effect of drug treatment on the peak amplitude and AUC of the baseline-corrected fluorescent signals in both calcium imaging experiments. 

### 4.10. Radioligand Binding Assays

All fractions and pure barbamide were sent to the National Institute of Mental Health’s Psychoactive Drug Screening Program for screening against a panel of 47 receptors including serotonin, dopamine, adrenergic, muscarinic, opioid and sigma receptors, along with monoamine transporters. Methods for these assays are provided online at https://pdsp.unc.edu/pdspweb/. For primary binding assays, the sample concentration was 10 µM, and any inhibition greater than 50% is considered a significant hit. The IC_50_ is reported rather than the K_i_ because, at the fraction level, multiple compounds of unknown molecular weight are present. The results of the full panel screen are available from the authors upon request. 

## Figures and Tables

**Table 1:** Results of preliminary receptor affinity screen for sub-fractions of DUQ0029. Values are average percent inhibition of receptors at 10 µM (N = 3). No SD is reported for these values as they are a primary screen for prioritization of fractions. 5-HT = serotonin receptors; KOR = kappa opioid receptor.

Fraction	5-HT1A	5-HT2A	5-HT2B	KOR	Sigma 1	Sigma 2 (*tmem97*)
DUQ0029A	40.2	−1.0	4.8	37.8	45.0	16.5
DUQ0029B	21.4	22.0	19.5	30.8	68.2	69.9
DUQ0029C	43.1	35.3	66.2	25.2	63.4	70.6
DUQ0029D	77.3	64.8	61.4	89.5	70.8	62.7
DUQ0029E	43.4	30.7	24.9	50.0	74.5	69.0
DUQ0029F	4.0	13.5	0.8	24.3	71.9	69.7
DUQ0029G	25.0	15.8	3.2	27.2	74.5	54.5
DUQ0029H	72.7	13.8	39.5	10.7	68.4	55.0
DUQ0029I	61.6	0.0	71.6	18.3	65.1	34.1

**Table 2:** Results of receptor affinity screen for barbamide and barbamide-containing fractions. Values are average percent inhibition of receptors at 10 µM (N = 3). No SD is reported for these values as they are a primary screen for prioritization of fractions. D = dopamine receptor; DAT = dopamine transporter.

Name	D1	D3	DAT	KOR	Sigma 1	Sigma 2
DUQ0029D	21.0	23.4	40.4	89.5	70.8	62.7
DUQ0032F3	67.5	24.1	41.6	93.3	28.5	73.0
DUQ0037F3	46.4	1.8	21.3	82.8	33.2	63.5
Barbamide	43.2	57.9	74.2	94.7	50.0	75.2

**Table 3:** Treatment with barbamide had a minimal impact on cell viability in mammalian cells after 72 h. Barbamide was tested at concentrations of 0.1, 0.3, 1, 3, 10, 30, and 100 M. Experiments were run in triplicate.

Cell Line	Cell Type	IC_50_ (µM)
MDA-MB-231	TNBC	40.7 ± 3.5
BT-549	TNBC	42.1 ± 5.1
MCF-7	Luminal A Breast Cancer	38.9 ± 8.1
HEK-293	Human Embryonic Kidney	51.6 ± 0.6

## Data Availability

The data that support the findings of this study are available from the corresponding authors upon reasonable request.

## Supplementary Materials

The following supporting information can be downloaded at: https://www.mdpi.com/article/10.3390/md21020110/s1, Figure S1. 1H NMR spectrum of barbamide (**1**) in CDCl3 at 500 MHz; Figure S2. 13C spectrum of barbamide (**1**) in CDCl3 at 500 MHz; Figure S3. COSY spectrum of barbamide (**1**) in CDCl3 at 500 MHz; Figure S4. HSQC spectrum of barbamide (**1**) in CDCl3 at 500 MHz; Figure S5. HMBC spectrum of barbamide (**1**) in CDCl3 at 500 MHz.
