# Supplementary material for: Barbamide Displays Affinity for Membrane-Bound Receptors and Impacts Store-Operated Calcium Entry in Mouse Sensory Neurons
Source: Mar Drugs. 2023 Feb 2;21(2):110. doi: 10.3390/md21020110 (PMC9966578; doi:10.3390/md21020110)
Supplement: Supplementary file 1 [file marinedrugs-21-00110-s001.zip › marinedrugs-2142422-supplementary.pdf]

Title: Barbamide binds to kappa opioid receptors and enhances store-operated calcium entry in mouse sensory neurons.

Authors: Andrea Hough[1], Connor Criswell[2], Asef Faruk,[1] Jane E. Cavanaugh,[1] Benedict Kolber[2,**], Kevin Tidgewell[1,**]

[1]Duquesne University, Division of Medicinal Chemistry, Pittsburgh, PA 15282; [2]University of Texas at Dallas, Department of Neuroscience, Center for Advanced Pain Studies Richardson, TX 75080;

Contents of Supporting Information

Contents	page
**Figure S1**. ^{1}H NMR spectrum of barbamide (**1**) in $CDCl_3$ at 500 MHz	**2**
**Figure S2**. ^{13}C spectrum of barbamide (**1**) in $CDCl_3$ at 500 MHz	**3**
**Figure S3**. COSY spectrum of barbamide (**1**) in $CDCl_3$ at 500 MHz	**4**
**Figure S4**. HSQC spectrum of barbamide (**1**) in $CDCl_3$ at 500 MHz	**5**
**Figure S5**. HMBC spectrum of barbamide (**1**) in $CDCl_3$ at 500 MHz	**6**

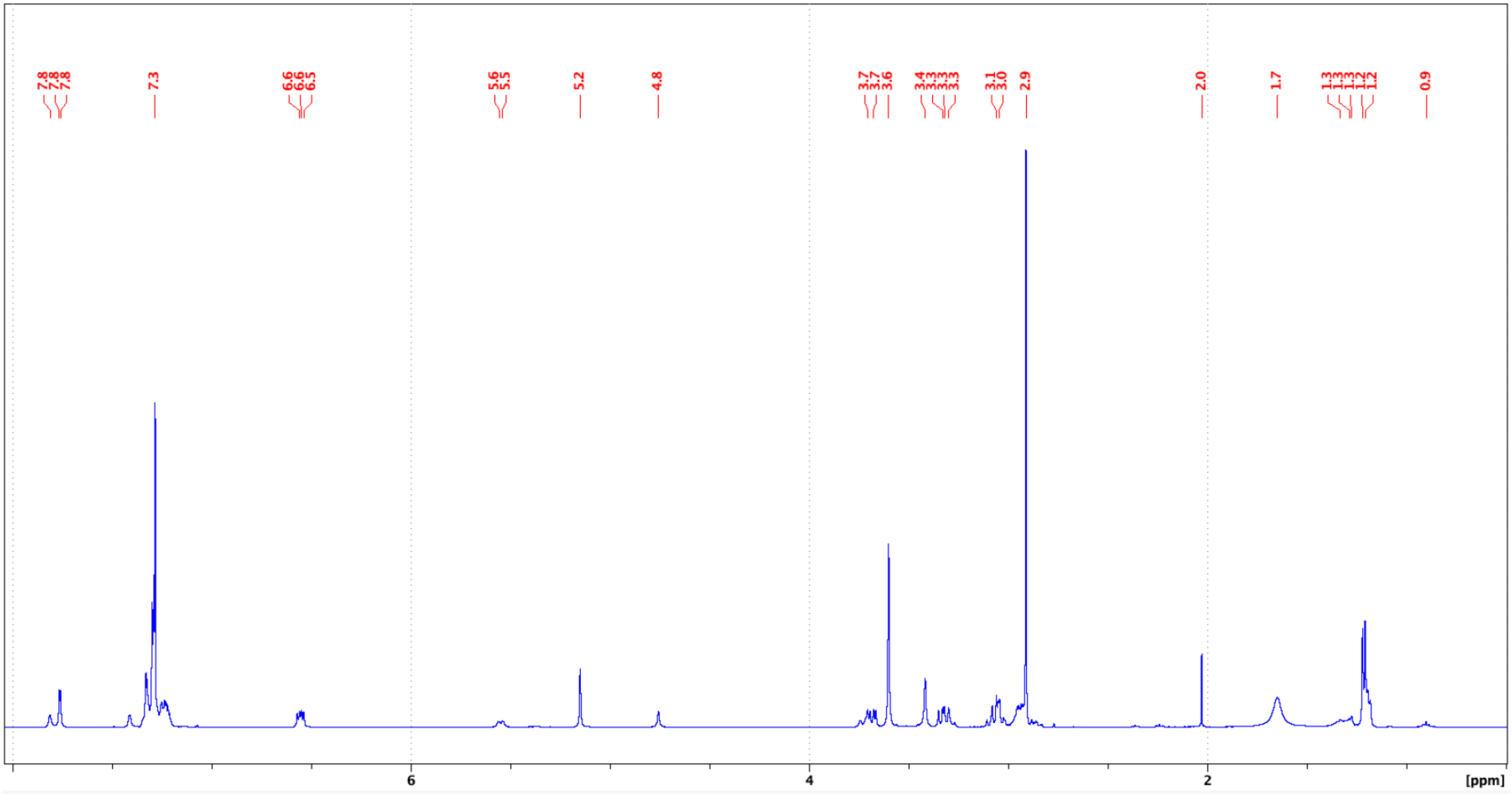

Figure S1. ^{1}H NMR spectrum of barbamide (**1**) in $CDCl_3$ at 500 MHz

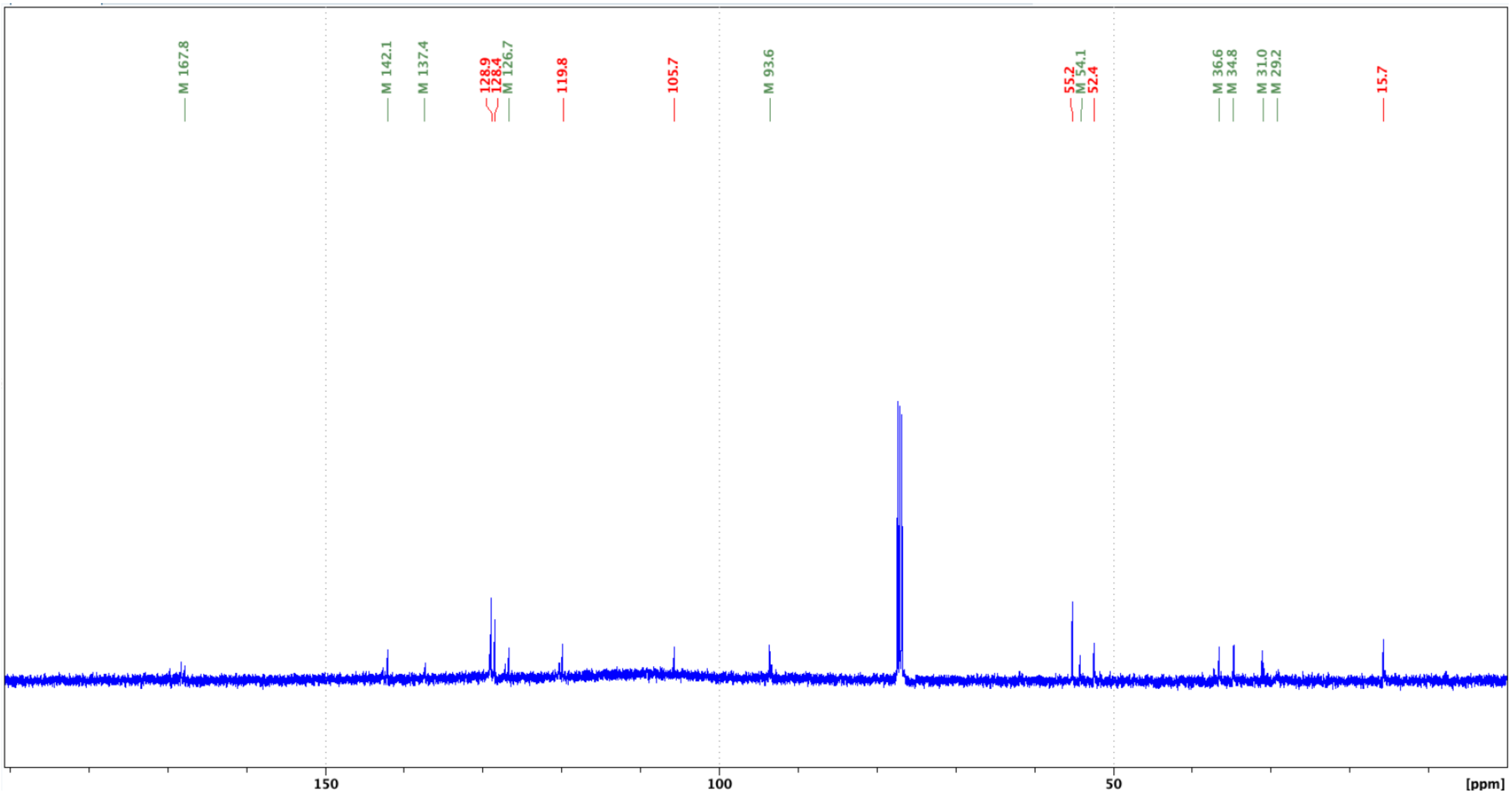

Figure S2. ^{13}C spectrum of barbamide (**1**) in $CDCl_3$ at 500 MHz

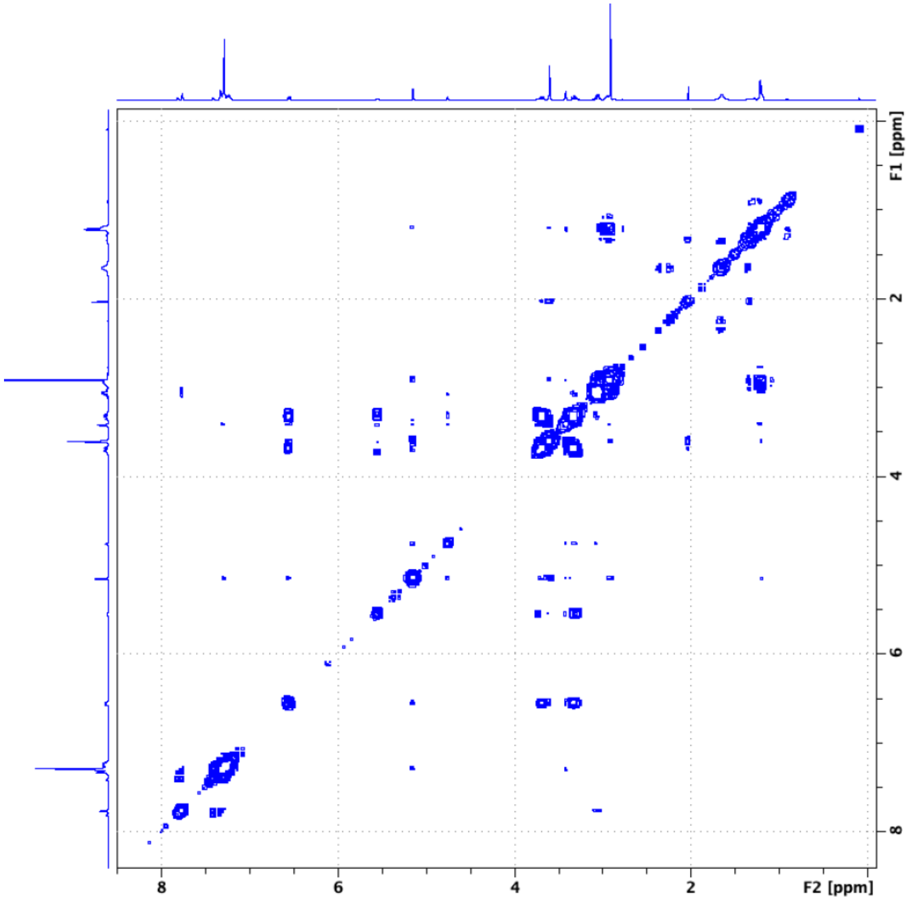

Figure S3. COSY spectrum of barbamide (**1**) in $CDCl_3$ at 500 MHz

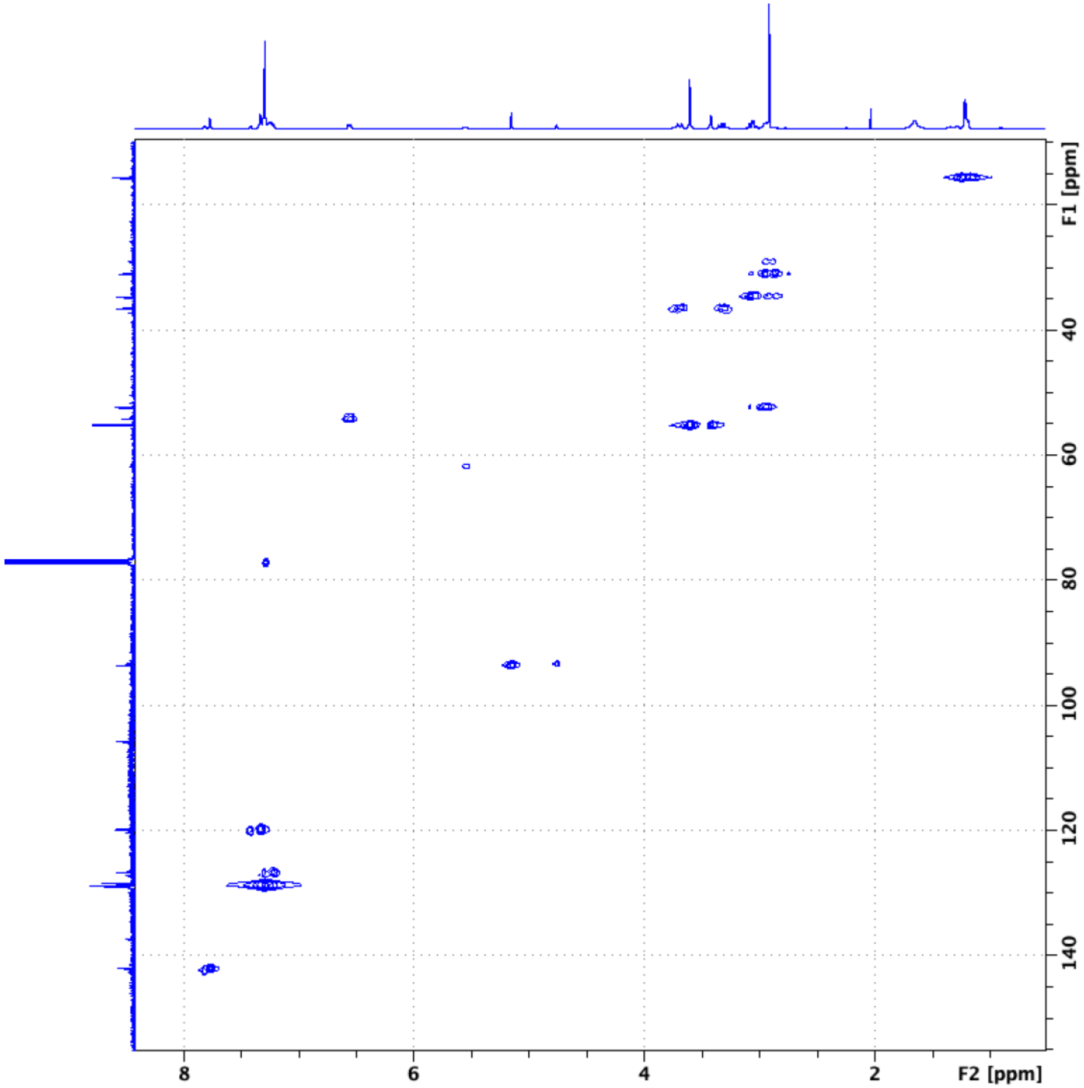

Figure S4. HSQC spectrum of barbamide (**1**) in $CDCl_3$ at 500 MHz

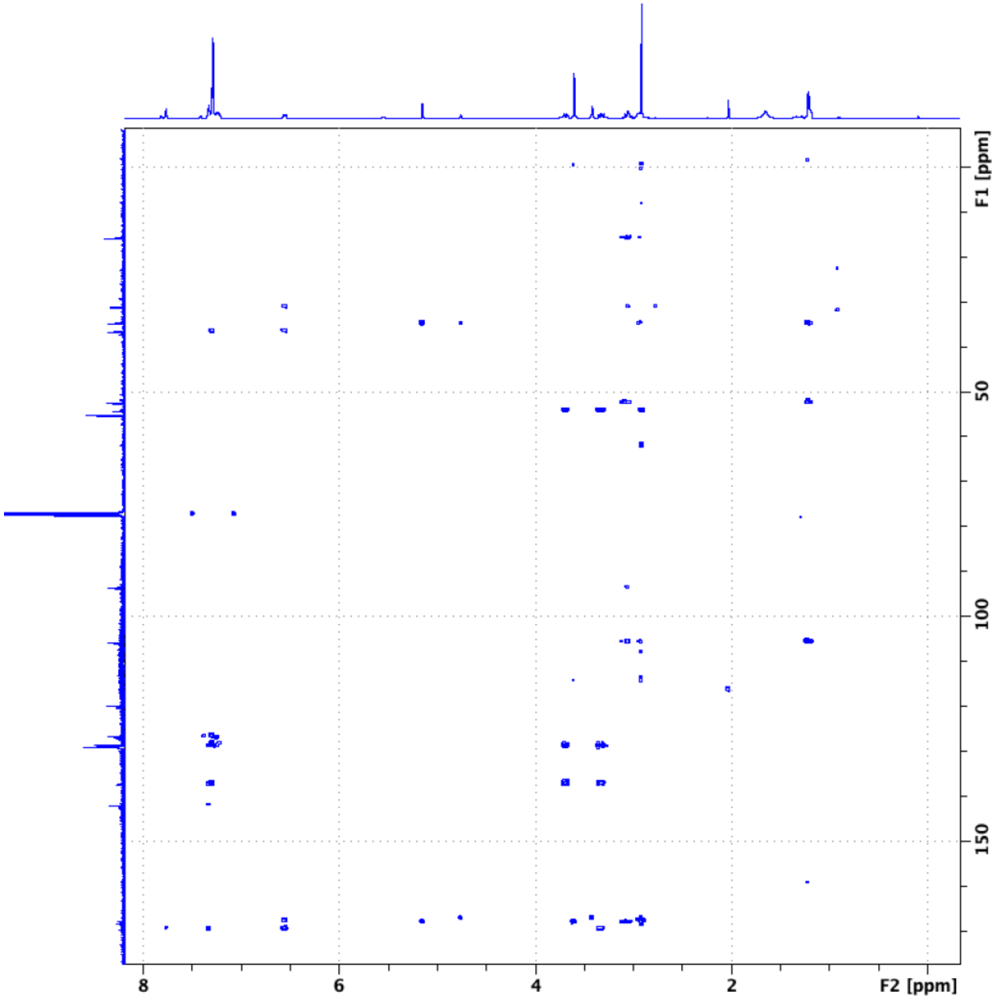

Figure S5. HMBC spectrum of barbamide (**1**) in $CDCl_3$ at 500 MHz
